# Melatonin and carcinogenesis in mice: the 50th anniversary of relationships

**DOI:** 10.18632/oncotarget.28537

**Published:** 2023-12-12

**Authors:** Vladimir N. Anisimov, Alexey G. Golubev

**Affiliations:** ^1^Department of Carcinogenesis and Oncogerontology, N.N. Petrov National Medical Research Center of Oncology, Saint Petersburg 197758, Russia; ^*^The authors contributed equally to this work

**Keywords:** cancer, melatonin, mice

## Abstract

The history of studies of melatonin effects on cancer in mice is outlined, the main lesson being that the systemic *in vivo* effects of melatonin on animals may overwhelm the *in vitro* effects found using tissue explants or cell cultures. In particular, the timing of melatonin administration is of crucial importance for using the drug, which is freely available over counter and thus needs no licensing for its applications in oncology.

## INTRODUCTION

Fifty years ago, in 1973, V. Anisimov and coauthors have demonstrated for the first time an inhibitory effect of the pineal gland hormone melatonin on cancer *in vivo*, namely on transplantable mammary tumors in mice [1]. Subsequently, it was shown in a number of studies that melatonin administration with drinking water at night inhibits chemically induced mammary carcinogenesis in mice and rats (reviewed in [2, 3]).

On the contrary, maintaining female mice and rats under round-the-clock lighting conditions, which suppresses the nighttime production of melatonin, stimulates spontaneous and chemical carcinogen-induced mammary tumors development [4].

**Figure 1:**
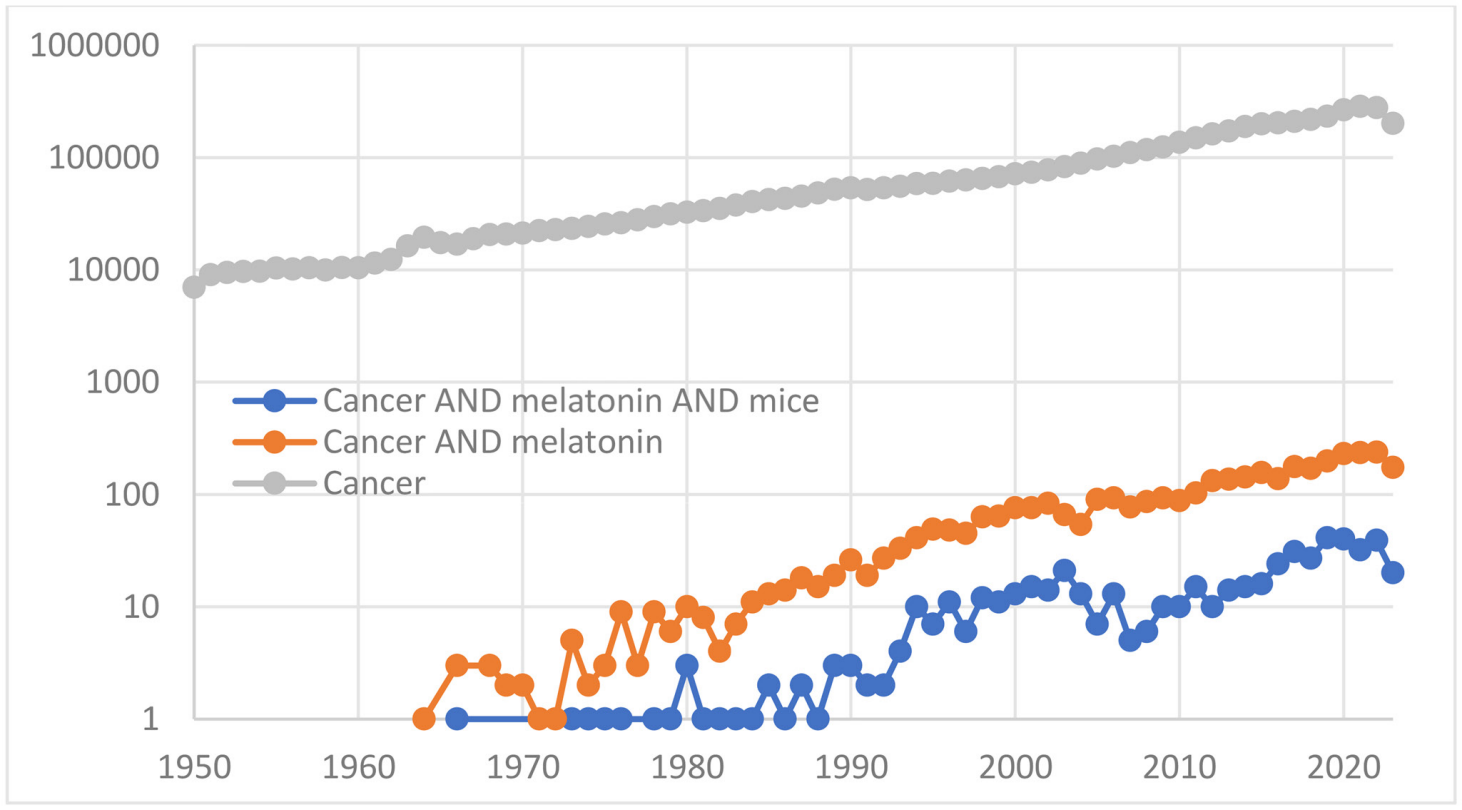
Trends in the annual numbers of publications.

As of today, the query “cancer AND melatonin AND mice” in Pubmed returns about 550 entries. In Figure 1 the time course of the annual numbers of such publications is compared with the time courses of publications on melatonin and cancer and on cancer in general. All trends are traced after 1958, the year of the discovery of melatonin. The reports on experiments with mice are obviously much less numerous than on melatonin and cancer in general (NB: Y-axis is logarithmic). Both trends related to melatonin are parallel to the trend of publications on cancer, and all three trends are almost exponential (linear in a semilogarithmic plot), as usual. However, the “cancer, melatonin and mice” trend is peculiar in that the interest in melatonin-and-cancer-related work with mice seems to be going down in the period from about 2005 to 2015; however, the interest rejuvenated thereafter. No reasons for this zigzag are apparent.

### Early studies

Other controversies, which were recognized as early as in 1970-ies, include the lack of consistency in the antitumor effects of melatonin observed in *in vivo* models, including murine ones, as has been analyzed by Bartsch and Bartsch in 1981 [5], see Table 1.

**Table 1:** Controversies in the early studies of the effects of melatonin on cancer in rodents (modified from ref. [5])

References	Experimental system	Results
***Tumor inhibition in intact animals***		
[1]	Transplantable mammary tumor in mice	Inhibition of tumor development
[6]	Solid leukemia in mice (i.m.)	Statistically significant inhibition of tumor size
[7]	Methylcholanthrene-induced sarcoma in mice	Slight delay in occurrence of tumors
[8]	Transplantable mammary tumor in rats	Statistically significant decrease in tumor size
[9]	DMBA-induced mammary tumors in rats	Decreased tumor incidence and tumor growth (melatonin given at 5 p.m.)
***Tumor inhibition in pinealectomized animals***		
[9]	DMBA-induced mammary tumors in rats	Decreased tumor incidence
[10]	Transplantable melanoma in hamsters	Inhibition of tumor development
[7]	Yoshida sarcoma in Wistar rats	Prolonged survival time
***No effect in intact animals***		
[7]	Yoshida sarcoma in Wistar rats	No effect
[11]	Yoshida sarcoma in rats	No effect
[12]	Walker carcinosarcoma in rats	No effect
[9]	DMBA-induced mammary tumors in rats	No effect under constant light
***No effect in pinealectomized animals***		
[9]	DMBA-induced mammary tumors in rats	No effect under constant light
***Tumor stimulation in intact animals***		
[13]	DMBA-induced mammary tumors in rats	Increased tumor incidence and higher degree of malignancy
[7]	Lewis lung carcinoma in mice	Slight reduction of survival time

To find the cause of the apparent discrepancies, mice were used by Bartsch and Bartsch [5]. These authors came to the important conclusion that the effects of melatonin on cancer crucially depend on the time of treatment: “It appears that under long photoperiods melatonin shows opposite effects on fibrosarcoma ascites and Ehrlich solid tumors depending on the time of the day at which the compound was administered. Tumors are stimulated by melatonin injections in the morning and inhibited by late afternoon injections.”

In more general terms, this conclusion means that the effects of melatonin depend on animal conditions and therefore, since melatonin is a hormone, which is able to change these conditions, melatonin effects on cancer must depend to a significant extent on the systemic effects of melatonin, which may be seen only in animal models, not in cell cultures, *ex vivo* explants etc.

### Murine models for melatonin-vs-cancer studies

Murine models for studying melatonin effects on cancer comprise, in the time order of their introduction, mice grafted with murine tumors [1], chemically induced tumors [7], spontaneous carcinogenesis in mice [14, 15], transgenic HER2/neu oncogene-bearing mice [16, 17], and nude mice grafted with human prostate tumors [18]. Besides mammary and prostate carcinomas, other cancers, such as tumors of the colon [19], lung [20], muscle [21], and stomach [22], were studied in mice under the influence of melatonin. An important aspect of such studies is that the effects of melatonin not only on cancer itself, but also on the consequences of anticancer therapy, including its side-effects, may be evaluated.

Each line of experimentation has provided for coming to important results and conclusions. For example, lifelong treatment of mice with melatonin decreased the incidence of spontaneous tumors, mostly mammary carcinomas, only at a low content of melatonin in drinking water (2 mg/L), but not at a high content (20 mg/L) [23]. The potentiating effect of melatonin on cytotoxic therapy against mammary tumors in HER2/neu transgenic mice was shown to depend on the timing of melatonin administration relative to cytotoxic drug administration [24]. Melatonin was shown to alleviate the depression syndrome in mice treated with the alkylating agent temozolomide used in therapy for brain cancer [25]. The ability of melatonin to alleviate the side effects of cytotoxic drugs and radiation has been demonstrated in several murine models, e.g., [26–28].

In nude mice grafted with human gastric cancer, melatonin inhibited lung metastases development, importantly, by suppressing the epithelial to mesenchymal transition (EMT) [22]. EMT was inhibited by melatonin in a murine model of low-grade inflammation [29]. Although no antitumor effects of melatonin were evaluated in this model, the increasing awareness of the importance of inflammation and EMT for primary cancer and metastases development [30] makes this finding highly relevant for oncology.

## CONCLUSION

Thus, having been used for fifty years, murine models proved to be valuable and, in some cases, indispensable for advancing melatonin applications in oncology by suggesting novel facets of melatonin effects and utility. Because the employment of melatonin beyond its established clinical fields needs no licensing, such suggestions may be tested in practice more readily than in the cases of other drugs. In fact, such trials have already yielded promising clinical results, e.g., [31].
